# Immunogenicity and Efficacy of a Measles Virus-Vectored Chikungunya Vaccine in Nonhuman Primates

**DOI:** 10.1093/infdis/jiz202

**Published:** 2019-05-03

**Authors:** Shannan L Rossi, Jason E Comer, Eryu Wang, Sasha R Azar, William S Lawrence, Jessica A Plante, Katrin Ramsauer, Sabrina Schrauf, Scott C Weaver

**Affiliations:** 1 Institute for Human Infections and Immunity, University of Texas Medical Branch, Galveston; 2 Department of Microbiology and Immunology, University of Texas Medical Branch, Galveston; 3 Institute for Translational Science, University of Texas Medical Branch, Galveston; 4 World Reference Center for Emerging Viruses and Arboviruses, University of Texas Medical Branch, Galveston; 5 Themis Bioscience GmbH, Vienna, Austria

**Keywords:** chikungunya virus, measles virus, nonhuman primate, vaccine

## Abstract

**Background:**

Chikungunya virus (CHIKV) infection can result in chikungunya fever (CHIKF), a self-limited acute febrile illness that can progress to chronic arthralgic sequelae in a large percentage of patients. A new measles virus-vectored vaccine was developed to prevent CHIKF, and we tested it for immunogenicity and efficacy in a nonhuman primate model.

**Methods:**

Nine cynomolgus macaques were immunized and boosted with the measles virus-vectored chikungunya vaccine or sham-vaccinated. Sera were taken at multiple times during the vaccination phase to assess antibody responses against CHIKV. Macaques were challenged with a dose of CHIKV previously shown to cause fever and viremia, and core body temperature, viremia, and blood cell and chemistry panels were monitored.

**Results:**

The vaccine was well tolerated in all macaques, and all seroconverted (high neutralizing antibody [PRNT_80_ titers, 40–640] and enzyme-linked immunosorbent assay titers) after the boost. Furthermore, the vaccinated primates were protected against viremia, fever, elevated white blood cell counts, and CHIKF-associated cytokine changes after challenge with the virulent La Reunión CHIKV strain.

**Conclusions:**

These results further document the immunogenicity and efficacy of a measles-vectored chikungunya vaccine that shows promise in Phase I–II clinical trials. These findings are critical to human health because no vaccine to combat CHIKF is yet licensed.

## Teaser

Chikungunya fever is a debilitating human disease. A measles-based vaccine expressing chikungunya proteins can protect nonhuman primates from disease, including fever, white blood cell count reduction, viremia, and proinflammatory cytokine levels. Lineage cross-reactive antibodies are also produced.

Chikungunya virus (CHIKV), an alphavirus in the *Togaviridae* family, is the etiologic agent of chikungunya fever (CHIKF), a febrile disease typically accompanied by rash and severe, debilitating, and often chronic arthralgia [1, 2]. Although the case-fatality rate is typically less than 1% and fatalities usually involve the elderly with comorbidities, perinatal CHIKV transmission from viremic women to infants can also result in fatal outcomes or severe neurologic sequelae. Attack rates are typically high during explosive outbreaks, sometimes involving more than half of a naive population, due in part to the dearth of inapparent infections compared, for example, with dengue, Zika, and yellow fever virus infections that can affect the same regions. Therefore, CHIKV has both a major public health and economic impacts because the chronic arthralgia, which can last for years, and affects the ability of victims to work and care for their families.

Chikungunya virus originated in an enzootic, nonhuman primate (NHP)-arboreal mosquito vector cycle in sub-Saharan Africa, but it has emerged and spread into Asia and the Americas [3, 4]. These emergences typically involve transmission by *Aedes* species mosquitoes with humans serving as amplification hosts. Similar emergence of dengue, yellow fever, and Zika virus into urban transmission cycles has been devastating to public health throughout the tropics and subtropics [2]. The only control measures typically available—reductions in mosquito populations or education to reduce contact of people with these vectors—have had little impact on human infections. In the case of CHIKV, the most recent urban emergences began in 2004 in Kenya [5] and spread (1) into islands of the Indian Ocean as well as (2) from Africa to India and to Southeast Asia. These epidemics resulted in exportation via viremic travelers to initiate outbreaks in Italy [6] and France [7], underscoring the ability of CHIKV to spread to nontropical areas. In 2013, CHIKV arrived in the Caribbean and caused a massive outbreak in completely naive populations of the Americas [8], followed closely behind in 2014 by the introduction into Brazil and spread of an additional strain from Africa [9]. Chikungunya virus transmission remains very active, especially in South America, and has recently reemerged in India, Pakistan, Italy, and France [2].

Because CHIKV antigenic diversity is limited and antibodies alone are typically sufficient to prevent disease caused by alphaviruses, vaccines are an attractive approach to control CHIKF and urban outbreak spread. Several CHIKV vaccine candidates have been described, including the use of almost all major platforms (G. Rezza and S. C. W., manuscript in preparation). Of these, 2 have advanced into Phase II clinical trials, including a recombinant, live-attenuated vaccine comprising the Schwarz vaccine strain of measles virus (MV) engineered to express the CHIKV structural proteins. This vaccine, MV-CHIK, is highly immunogenic and protects mice from lethal CHIKV challenge [10]. In a randomized, double-blind, placebo-controlled, Phase I clinical trial with 3 different vaccine doses, seroconversion rates of 44%–92% occurred after single doses and reached 100% after a second immunization. The safety profile of MV-CHIK was good with no serious adverse events associated with vaccination [11]. More importantly, prior measles immunity did not appear to affect the immunogenicity of this CHIKV vaccine. These promising results supported the advancement of MV-CHIK into Phase II Clinical trials NCT02861586 and NCT02861586 (clinicaltrials.gov). The Phase II Clinical trial NCT03101111 performed in Europe was completed and the results were recently described [12]. The study confirmed the good safety, tolerability, and immunogenicity of the vaccine candidate, independent of pre-existing immunity against the vector. A single immunization induced neutralizing antibodies in up to 93% of participants, and a second vaccination induced high titers with seroconversion rates of up to 100% in all MV-CHIK treatment groups.

Final preclinical evaluation of alphavirus vaccines typically relies on NHP models, which closely mimic human infections. Therefore, to further evaluate the efficacy of MV-CHIK, we used cynomolgus macaques (*Macaca fascicularis*), one of the optimal models of human CHIKV infection, in vaccination and challenge studies. After subcutaneous challenge, these animals developed fever and viremia similar to human infections [13–16]. Therefore, they are considered to be an ideal model for demonstrating vaccine efficacy before proceeding into pivotal Phase III clinical trials for licensure. In this study, we vaccinated cynomolgus macaques with MV-CHIK and demonstrated strong immunogenicity as well as protection from all measures of disease and viremia after challenge with wild-type CHIKV.

## MATERIALS AND METHODS

### Nonhuman Primates

Adult cynomolgus macaques (4 males and 5 females) weighing 2.7–3.2 kg and determined to be alphavirus antibody-free based on hemagglutination inhibition assays for western, eastern, and Venezuelan equine encephalitis viruses, CHIKV, Sindbis, and Semliki Forest viruses, and MV were obtained from Covance (Alice, TX). The animals were quarantined for 14 days. Housing consisted of individual open metal caging units that allowed visual inspection and prevented contact with other animals in the room. Nonhuman primates were maintained on a 2050 Teklad Global 20% Protein Primate Diet. For enrichment, animals were fed an additional food item (fruit, vegetable, or other edible treat) at least once daily as well as various forms of manipulanda such as plastic or rubber balls, Kong toys, mirrors, etc. A veterinarian conducted a physical examination of each animal during the quarantine period to ensure the animals’ health and implanted the animals with TA10TA-D70 telemetry transponders (Data Sciences International, St. Paul, MN).

Animals were observed twice daily upon arrival, and observations requiring manipulation were performed under anesthesia. Cage-side observations were performed before administering anesthesia and after the animals had recovered. Clinical observations were recorded throughout the study, and body weights were recorded during each physical exam and on study days 28, 56, 57, and 60. Body temperatures and activity were measured via implanted telemetry transponders from study day 1 to 77. During the vaccination phase, measurements were recorded every 10 minutes. The recording frequency was increased to every 60 seconds for the initial 7 days postchallenge, but it was then returned to every 10 minutes for the remainder of the study. The activity, expressed as counts per minute, is based on the signal strength between the transponders and their respective receivers. The signal strength changes as the location of the transponders changes relative to the receivers to which they are linked, and these changes in signal strength are interpreted as counts and denote movement. All temperature and activity data are presented as 2-hour moving averages.

Blood samples were collected from sedated animals into serum separator tubes and tubes containing ethylenediaminetetraacetic acid (EDTA) on study days 0, 28, and 56–60. Whole blood collected in the EDTA tube was analyzed for the parameters listed in Table 1 using a HEMAVET multispecies hematology instrument (Drew Scientific, Dallas, TX). Serum was analyzed on a VetScan VS2 Chemistry Analyzer (Abaxis, Union City, CA) for the parameters specified in Table 2.

**Table 1.** Reciprocal PRNT Titers After Vaccination/Boost

Group	NHP ID	Day 0	Day 28 PRNT_80_	Day 28 PRNT_50_	Day 56 PRNT_80_	Day 56 PRNT_50_
MV-CHIK-Vaccinated	13362	<20	<20	160	>640	>640
	13367	<20	20	320	>640	>640
	13365	<20	20	160	160	>640
	13426	<20	40	160	>640	>640
	13422	<20	40	>640	40	>640
	13421	<20	160	160	>640	>640
Sham-Vaccinated	13428	<20	<20	<20	<20	<20
	13424	<20	<20	<20	<20	<20
	13368	<20	<20	<20	<20	<20

Abbreviations: CHIKV, Chikungunya virus; ID, identification; MV, measles virus; NHP, nonhuman primate; PRNT, plaque reduction neutralization test.

**Table 2.** Reciprocal Cross-Neutralization (PRNT_50_) Against Several CHIKV Lineages

Group	NHP ID	LR (Indian Ocean)	SV-0444-95 (Asian)	37997 (West African)	YO111213 (Asian/American)
NHP MV-CHIK-Vaccinated	13362	>640	>640	>640	>640
	13367	NR^a^	NR^a^	>640	20
	13365	320	160	>640	20
	13426	NR^a^	160	>640	40
	13422	40	80	>640	80
	13421	NR^a^	NR^a^	NR^a^	NR^a^
NHP Sham-Vaccinated	13428	<20	<20	<20	<20
	13424	<20	<20	<20	<20
	13368	<20	<20	<20	<20
Human Pool 1^b^	N/A	640	160	>640	>640
Human Pool 2^b^	N/A	<40	20	80	40

Abbreviations: CHIKV, Chikungunya virus; ID, identification; LR, La Reunión; MV, measles virus; N/A, not applicable; NHP, nonhuman primate; NR, not run; PRNT, plaque reduction neutralization test.

^a^Not run due to low sample volume.

^b^Pooled sera from a Phase I clinical trial of MV-CHIK. Pool 1 CHIKV PRNT_50_ high titer; Pool 2 CHIKV PRNT_50_ low titer.

All experiments and procedures were approved by the UTMB Institutional Animal Care and Use Committee. UTMB is an Association for Assessment and Accreditation of Laboratory Animal Care International-accredited facility.

### Viruses and Assays

The MV-vectored vaccine has been described previously [10], as well as the CHIKV challenge La Reunión (LR) strain; strains YO111213, SV0444-95, and 37997 were obtained from the World Reference Center for Emerging Viruses and Arboviruses at UTMB [17, 18]. The MV-CHIK vaccine was shipped to UTMB as a ready-to-use lyophilized good manufacturing practice product with a titer of 5 × 10^5^ (±0.5 log) median tissue culture infectious dose (TCID_50_)/vial. The same material was also used in the Phase II clinical trial in Europe (clinical trial NCT02861586). Before immunization, the lyophilized freeze-dried pellet was reconstituted with 0.4 mL sterile water for injection.

The MV-CHIK titers were determined by TCID_50_ assay on Vero cells 10–87 (American Type Culture Collection [ATCC], Manassas, VA) using standard methods. In brief, Vero cells were seeded in 96-well plates and infected 1 day later by a 5-fold serial dilution of MV-CHIK. This endpoint dilution assay quantifies the amount of virus required to kill 50% of infected host cells or to produce a cytopathic effect in 50% of inoculated tissue culture cells. The TCID_50_ was calculated by the Spearman/Kärber method 6 days postinfection.

Chikungunya virus titers were determined by plaque assay on Vero cells (ATCC) using standard methods [19] and by quantitative real-time polymerase chain reaction assays (RT-qPCR). Ribonucleic acid (RNA) was isolated from whole blood using a viral RNA mini prep kit (QIAGEN). The presence of CHIKV genomic RNA was confirmed using virus-specific primers and probe (CHIK, 243F GAY CCC GAC TCA ACC ATC CT; CHIK, 330R CAT MGG GCA RAC GCA GTG GTA; CHIK, 273P 6FAM AGY GCG CCA GCA AGG AGG AKG ATG T) using the Bio-Rad (Hercules, CA) iTaq Universal Probes One-Step kit. Each sample was tested in duplicate, and average cycle threshold (Ct) values were extrapolated to plaque-forming unit equivalents (PFUe)/mL from a standard curve generated from serially diluted RNA prepared from CHIKV-LR that was previously tittered by plaque assay.

### Serology

Antibody responses were assayed using plaque reduction neutralization tests (PRNT_80_ and PRNT_50_) using standard methods [19]. Unneutralized samples yielded 59 CHIKV-LR PFU/well for immunogenicity studies and 26, 68, 25, 28 plaques/well for LR, SV0444-95, 37997, YO111213, respectively, for cross-neutralization studies. Antibody titers were recorded as the highest dilution of serum that inhibited 80% or 50% of plaque formation, respectively. Samples with reciprocal PRNT_50_ titers ≥20 were considered positive. Limits of detection are between 20 and 640, and any samples without a detectable titer were listed as either <20 or >640. Enzyme-linked immunosorbent assays (ELISA) were performed using an Eilat/CHIKV chimeric virus as described previously [20].

### Cytokine Analyses

Circulating cytokine levels were measured using a multiplex bead assay according to the manufacturer’s instructions (Thermo Fisher Scientific, Waltham, MA). Sera were analyzed in triplicate for the following analytes following the manufacturer’s instructions: epidermal growth factor, eotaxin, basic fibroblast growth factor, granulocyte colony-stimulating factor (G-CSF), granulocyte macrophage-CSF (GM-CSF), hepatocyte growth factor, interferon (IFN)-γ, interleukin (IL)-1β, IL-1RA, IL-2, IL-4, IL-5, IL-6, IL-8, IL-10, IL-12, IL-15, IL-17, interferon-γ-inducible protein-10 (IP-10), interferon-inducible T-cell alpha chemoattractant, monocyte chemoattractant protein-1 (MCP-1), macrophage-derived chemokine, migration inhibitory factor (MIF), monokine induced by IFN-γ, macrophage-inflammatory protein (MIP)-1α, MIP-1β, RANTES, tumor necrosis factor-α, and vascular endothelial growth factor (VEGF).

### Vaccinations and Challenges

On study days 0 and 28, 6 NHPs, including 3 males and 3 females, were anesthetized and vaccinated intramuscularly in the quadriceps muscles. The lyophilized vaccine, received in a 1-dose vial, was reconstituted with 0.4 mL sterile water and 0.35 mL was used for the vaccination. Three negative control animals received 0.35 mL phosphate-buffered saline (sham-vaccination). On study day 56, NHPs were challenged with 1.4 × 10^5^ PFU of CHIKV strain LR via the subcutaneous route.

### Statistical Analyses

Mixed analysis of variance was performed on ELISA absorbance values, white blood cell (WBC) counts, log_10_-transformed titer data, and log_2_-tranformed PRNT data. Data points that were below the lower limit of detection were assumed to be one half of the limit of detection for the purposes of graphing and statistical analyses. Likewise, data points that were above the upper limit of detection were assumed to be twice the limit of detection. All statistical analysis was performed with SPSS, version 25 (IBM Corp., Armonk, NY).

## RESULTS

### Immune Responses

To measure immune responses after vaccination, PRNT_80_ assays were performed on sera collected on study days 0 (day of first vaccination), 28 (before boost), and 56 (after boost). All NHPs vaccinated with MV-CHIK showed neutralizing titers ≥40 as measured by PRNT_80_ after 2 vaccine doses, and 5 of 6 seroconverted after 1 dose (Table 1). The effect of vaccination was statistically significant whether the result was measured as PRNT_50_ (*F*_(1,7)_ = 259.8, *P* < .001) or PRNT_80_ (*F*_(1,7)_ = 22.6, *P* = .002) where *F*_(x,y)_ is the *F* statistic with the associated degrees of freedom and *P* is the resultant *P* value, with the MV-CHIK vaccine resulting in higher neutralizing antibody titers than sham vaccination. Time postvaccination was also a significant factor for both PRNT_50_ (*F*_(1,7)_ = 72.8, *P* < .001) and PRNT_80_ (*F*_(2,14)_ = 11.0, *P* = .001), with neutralizing antibody titers generally increasing over time.

To determine the level of cross-reactive neutralizing antibodies against other lineages of CHIKV, day 56 sera were assayed by PRNT_50_ against CHIKV strains representing all major lineages, including SV-0444-95 (Asian lineage), 37997 (West African lineage), and YO111213 (Asian/American lineage) for all primates except 13421 (due to sample volume limitation) (Table 2). Every NHP LR-positive sample was also seropositive against the other CHIKV strains, suggesting that the MV-CHIK vaccine could elicit cross-protective antibody-mediated immunity against multiple strains representing the known CHIKV antigenic diversity. In addition, pools of serum collected from human subjects as part of a Phase I clinical trial in Austria [11] (EudraCT: 2013-001084-23) were also tested for cross-reactivity in the same PRNT_50_ assay. The CHIKV strain used in the PRNT assay did not significantly affect the outcome of the assay (*F*_(3,12) _= 2.3, *P* = .127), but the vaccination status of the NHP did (*F*_(1,4) _= 24.5, *P* = .008). This analysis confirmed the presence of cross-reactive human antibodies after MV-CHIK immunization, further emphasizing the cross-protective potential elicited by the vaccine candidate.

**Figure 1.**
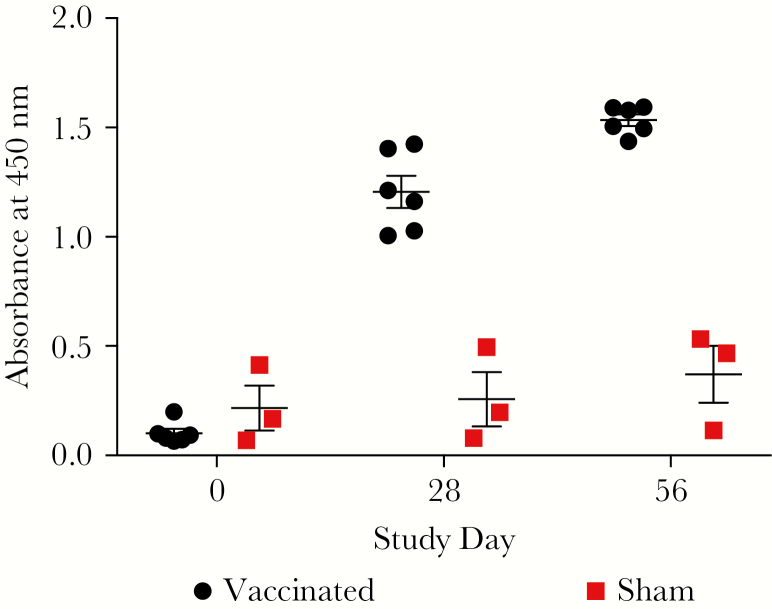
Anti-Chikungunya virus immunoglobulin titers in sera collected on days 0, 28, and 56 of the study as assayed by enzyme-linked immunosorbent assay. Sera yielding absorbance values that exceeded the means of negative control sera by more than 2 standard deviations were considered positive. The mean absorbance of the negative control sera was 0.08 at 450 nm. Error bars denote standard error.

The humoral response to NHP vaccination was also measured by ELISA. Figure 1 illustrates the relative anti-CHIK immunoglobulin (Ig)G responses of MV-CHIK-vaccinated versus control animals. All MV-CHIK-vaccinated animals were positive for anti-CHIKV IgG before challenge. Vaccination produced a statistically significant effect (*F*_(1,7)_ = 163.0, *P* < .001), as did time (*F*_(2,14)_ = 55.7, *P* = .000) and the vaccination/time interaction (*F*_(2,14)_ = 38.9, *P* < .001).

### Clinical Observations

**Figure 2.**
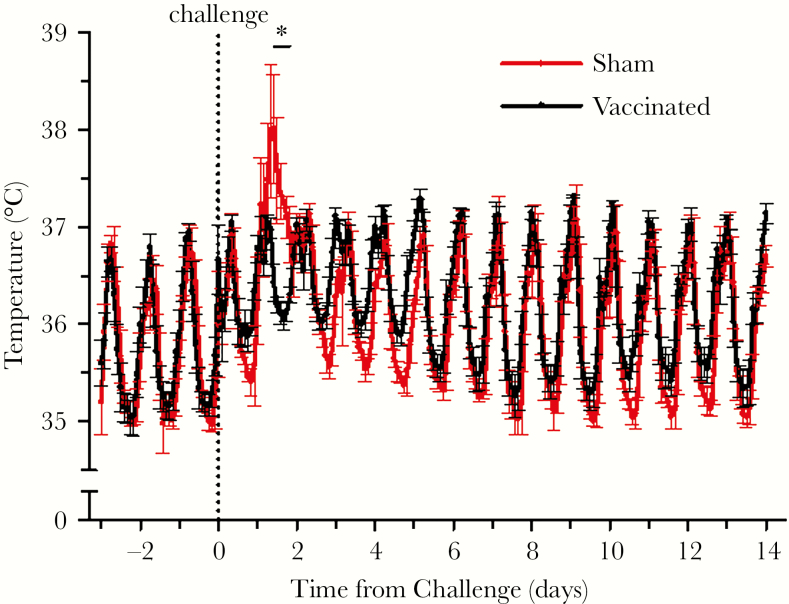
Body temperatures generated using a 2-hour moving average obtained from telemetric data. The vaccinated versus sham-vaccinated groups were compared statistically by one-way analysis of variance followed by Tukey’s test at each 2-hour time point beginning from 24 to 48 hours postchallenge. The mean temperature of the sham-vaccinated animals was significantly (*P* < .05) higher between 34 to 44 hours postchallenge as indicated by the asterisk.

**Figure 3.**
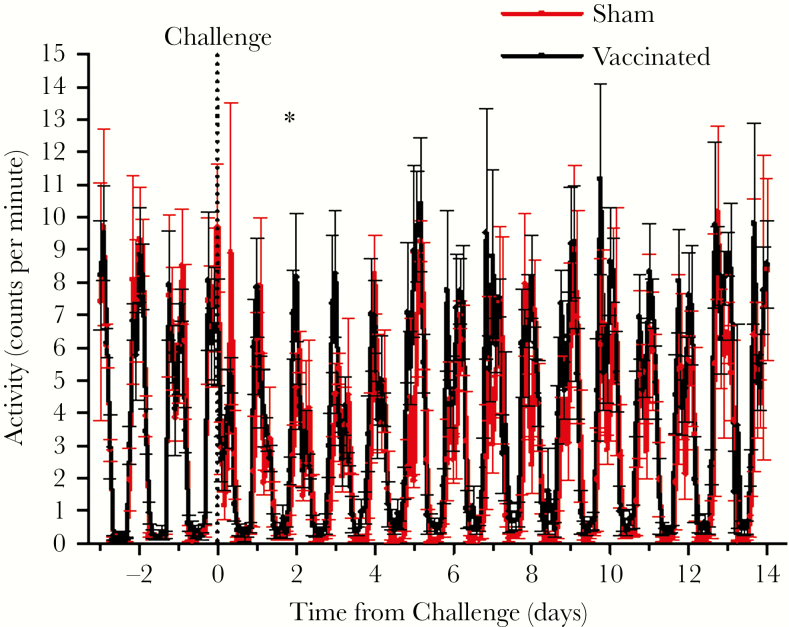
Nonhuman primate activity curves were generated using a 2-hour moving average. The vaccinated versus sham-vaccinated groups were compared statistically by one-way analysis of variance followed by Tukey’s test at each 2-hour time point beginning from 42 to 90 hours postchallenge. The mean activity of the sham-vaccinated animals was lower (*P *< .05) at hour 44 postchallenge as indicated by the asterisk. There are no units for activity.

There were no overt clinical signs of disease during any visual observations, including after vaccination (and at the vaccination site) and challenge. In addition, the body weights of all NHPs remained stable throughout the study. Body temperature (Figure 2) and activity (Figure 3) were monitored continuously during the challenge portion of the study, with baselines established using data collected on the 3 days before challenge. Figure 2 shows the normal diurnal periodicity of temperature with peaks in the late afternoon and lowest temperatures during the early morning. Transient fever was observed in the sham-vaccinated controls after challenge is seen as a spike beginning approximately 24 hours after challenge and lasting approximately 1 day. The significant increase in body temperature of up to approximately 1.5–2°C lasted for 10 hours, then the NHPs returned to normal temperatures and diel cycling. Monitoring of animal activity showed a significant difference between vaccinated and sham-vaccinated animals only at 1 time window: at the 44th hour of postchallenge, the former were more active than the latter (Figure 3).

### Hematology and Clinical Chemistry

Blood samples were analyzed for hematological changes on days 56–60. There were lower mean total WBC counts in the sham-vaccinated animals starting 2 days after challenge (Supplemental Figure S1) compared with vaccinated monkeys, with a trend toward significance based on vaccination status (*F*_(1,7)_ = 4.0, *P* = .086). Sham-vaccinated NHPs showed neutropenia and monocytopenia starting 2 days after challenge and also had decreased levels of lymphocytes on day 57 compared with NHPs receiving the MV-CHIK vaccine. Individual NHP results are listed in Supplemental Table 1. Clinical chemistry assays performed on sera collected on study days 56, 58, and 60 revealed no meaningful differences between control and vaccinated NHPs (Supplemental Table 2).

### Viremia

**Figure 4.**
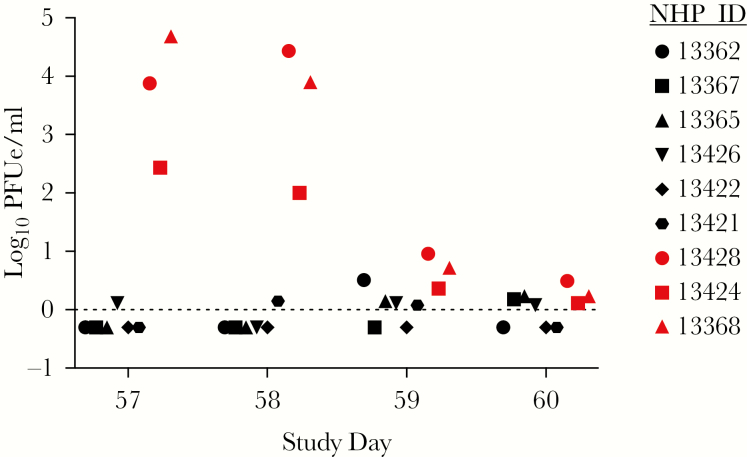
Viremia measured by quantitative real-time polymerase chain reaction assays. Ribonucleic acid (RNA) was isolated from sera and assayed using Chikungunya virus La Reunión (CHIKV-LR)-specific primers and probe. Cycle threshold (Ct) values were converted to plaque-forming units per milliliter (PFU/mL) from a standard curve generated from serially diluted RNA prepared from CHIKV-LR that was titered by plaque assay. Black symbols represent MV-CHIK vaccinated nonhuman primates (NHPs), whereas the red symbols indicate sham-vaccinated control animals.

Chikungunya virus titers in NHP blood were measured 1–4 days after challenge by RT-qPCR and plaque assay. All 3 sham-vaccinated animals became viremic, with mean titers of 1.9 × 10^4^, 1.2 × 10^4^, and 5.5 × 10^4^ PFUe/mL on study days 57, 58, and 59, respectively, as measured by RT-qPCR (Figure 4). By day 60, viremia levels were very close to or below the limit of detection (LOD <1 PFU/mL). The titers in NHPs vaccinated with MV-CHIK remained below or very close to the LOD over all sampling days. Overall, MV-CHIK-immunized animals had significantly lower serum CHIKV RNA titers than sham-vaccinated animals on study days 57–60 (*F*_(1,7)_ = 64.5, *P* = .000). Sera were also titered by plaque assay; NHPs 13428 and 13368 had 3.7 × 10^3^ and 1.3 × 10^4^ PFU/mL on day 57, respectively, and 1.9 × 10^3^ and 2.0 × 10^2^ PFU/mL on day 58, respectively (Supplemental Figure S2). Plaques were not detected in NHP 13424, which had the lowest titer via the more sensitive qRT-PCR assay. Immunized animals had significantly lower infectious CHIKV titers than sham-vaccinated animals on study days 57–60 (*F*_(1,7)_ = 9.2, *P* = .019).

### Circulating Cytokine Levels

**Figure 5.**
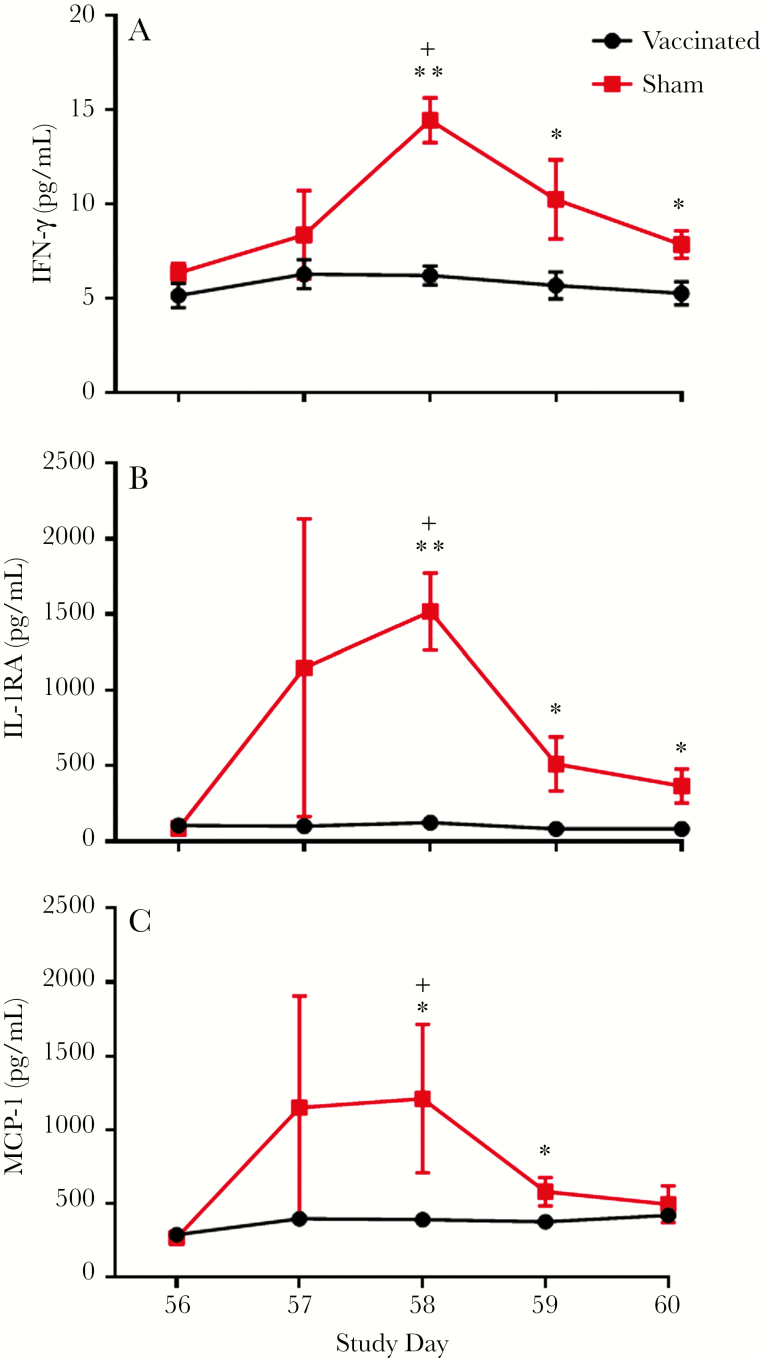
Mean levels of circulating cytokines in nonhuman primate serum assayed using the Monkey Cytokine Magnetic 29-Plex Panel (Invitrogen). Mean levels of interferon (IFN)-γ (A), interleukin (IL)-1RA (B), and monocyte chemoattractant protein-1 (MCP-1) are presented. One-way analysis of variance followed by Tukey’s multiple group comparisons were performed on the different time points for a given group; +, *P* < .05. Differences between groups were determined using an unpaired *t* test; *, *P* < .05 and **, *P* < .001. Bars indicate standard errors.

Cytokines in the sera collected on study days 56–60 were measured by ELISA. Three cytokines of the panel were significantly increased after challenge in sham-vaccinated animals compared with MV-CHIK-vaccinated NHPs. Circulating IFN-γ increased from 6.3 pg/mL just before challenge to 14.4 pg/mL on day 58 in the control group (*P* < .05, Tukey test). The level of IFN-γ was higher in the control group compared with the MV-CHIK-vaccinated group on day 58 (*P* < .001, unpaired *t* test) and the levels remained higher (*P* < .05, unpaired *t* test) (Figure 5A). There were also increases in IL-1RA and MCP-1 on day 58 compared with day 56 (*P* < .05, Tukey test) in the sham controls. Interleukin-1RA was significantly higher in control animals when compared with MV-CHIK-vaccinated NHPs on days 58 (*P* < .001, unpaired *t* test), 59 and 60 (*P* < .05, unpaired *t* test) (Figure 5B). The MCP-1 was also significantly higher in control NHPs compared with vaccinated animals on days 58 and 59 (*P* < .05, unpaired *t* test) (Figure 5C).

## DISCUSSION

Many vaccines that have improved human health are live-attenuated versions such as the Schwarz and related attenuated strains of MV used for several decades in routine childhood immunization [21]. The advantages of live-attenuated vaccines include the induction of rapid, robust, and long-lived immunity, without the need for adjuvants. Although several new live-attenuated vaccines for CHIKF based on rational attenuation of the CHIKV genome have been described [17, 22, 23], these will require more extensive preclinical safety testing to ensure that attenuation is robust and stable. In contrast, live-attenuated vaccines produced by expression of target virus antigen genes from a viral vector such as MV, with a long history of safety in human use, may offer a faster and more efficient route to licensure. Thus, MV-CHIK was developed to capitalize upon the extensive safety record of the measles vaccine as well as the proven ability of the virus to express foreign genes in a highly immunogenic manner [24].

Our safety, immunogenicity, and efficacy results with cynomolgus macaques corroborate the findings of prior studies with mice [10] as well as those of Phase I and II clinical trials [11, 12]. Consistent with the lack of significant reactogenicity in clinical trials, the macaques we tested showed no signs of disease after vaccination. Almost all animals developed detectable neutralizing antibodies within 4 weeks of a single MV-CHIK vaccine dose, and all developed PRNT_80_ titers of ≥40 within 4 weeks of a second immunization. Comparable neutralizing antibody titers are highly correlated with protection against alphavirus infections in Investigational New Drug use, as well as using a wide variety of animal models [25]. In addition, antibodies elicited by MV-CHIK also showed cross-neutralizing potential against other CHIKV strains representing all known genetic and antigenic diversity, including SV-0444-95 (Asian lineage), 37997 (West African lineage), and YO111213 (Asian/American lineage). This was in good accordance with data from human sera collected from a MV-CHIK Phase I clinical trial [11] and analyzed by the same assay, showing the induction of cross-neutralizing antibodies. In total, these results suggest that the MV-CHIK vaccine induces solid, cross-protective antibodies, further highlighting the strong potential of this vaccine candidate.

The cross-reactivity of CHIKV antibodies was explored in Langsjoen et al [18]. Vaccination with an Indian Ocean lineage vaccine protected mice and nonhuman primates against Caribbean CHIKV disease and viremia, but cross-reactive PRNT assays were not performed. Serology suggests is only 1 serotype of CHIKV and antibodies produced against 1 lineage recognize another [26], and the minimal protective PRNT titer is anticipated to be 10 [27]. Our results, while showing cross-neutralization, produced a wide range of titers (20 to >640). Although these changes are not statistically significant, they varied widely. This may be due to the PRNT assay or a property of the viruses chosen. More work is needed to explore the lineage-specific breadth of CHIKV vaccine immune responses, especially for the West African lineage.

To confirm efficacy, we challenged the macaques with wild-type LR-CHIKV at a dose shown previously to generate consistent fever and viremia [14, 16]. Three sham-vaccinated animals developed ca. 1.5–2°C fever lasting approximately 1 day (Figure 2), viremia of 3–4 days duration peaking at 10^2^–10^5^ PFU/mL (Figure 5), and a transient reduction in activity (Figure 3). In contrast, no fever or reduction in activity and a barely detectable viremia were detected in vaccinated animals after challenge. Vaccination protected against leukopenia (Supplemental Figure S1) and an increase in several circulating proinflammatory cytokines after challenge (Figure 5).

Cytokines that are upregulated during acute human infection include IL-6, IL-16, IL-17, IP-10, MCP-1, MIF, stromal cell-derived factor-1α, IL-1rα, IL-2rα, G-CSF, GM-CSF, VEGF, IL-7, IL-12p40, and IFN-α2 [28–30]. Many murine studies also have shown that a strong innate immune response is critical for controlling acute CHIKV infection. Some cytokines were elevated in our sham-vaccinated NHPs, including MCP-1 and IL-1. It is interesting to note that other proinflammatory cytokines, including IL-6 and IL-10, were not elevated significantly in this study. This may reflect the difference between human and NHP CHIKF.

This study focused on the antibody response in nonhuman primates after vaccination. However, the individual components of the cellular immune response, mainly the contribution of T cells, was not explored. In addition, this study did not examine the length of the immune response, which would be a critical step moving forward.

## CONCLUSIONS

In conclusion, we report findings of safety, strong immunogenicity, and robust efficacy of MV-CHIK in a NHP model of human disease. Together with clinical Phase I and II studies in approximately 300 subjects, our results indicate that the MV-CHIK vaccine is safe, immunogenic, and efficacious and worthy of further development towards licensure.

## SUPPLEMENTARY DATA

Supplementary materials are available at *The Journal of Infectious Diseases* online. Consisting of data provided by the authors to benefit the reader, the posted materials are not copyedited and are the sole responsibility of the authors, so questions or comments should be addressed to the corresponding author.

jiz202_suppl_Supplementary_Figures

jiz202_suppl_Supplementary_Tables

## Notes

***Acknowledgments.*** We thank Colm Atkins, Terry Juelich, and Alexander Freiberg for sample preparation and assistance with the Bio-Plex assays and Douglas Brining along with the Animal Resources Center staff for performing the telemetry implantation surgeries and for dedicated service in nonhuman primate monitoring.

***Financial support*.** This research was funded by Themis Bioscience and by Grant AI120942 from the National Institutes of Health.

***Potential conflicts of interest.*** K. R. and S. S. are employees of Themis Bioscience. All authors have submitted the ICMJE Form for Disclosure of Potential Conflicts of Interest. Conflicts that the editors consider relevant to the content of the manuscript have been disclosed.
