# Supplementary material for: Immunogenicity and Efficacy of a Measles Virus-Vectored Chikungunya Vaccine in Nonhuman Primates
Source: J Infect Dis. 2019 May 3;220(5):735–42. doi: 10.1093/infdis/jiz202 (PMC6667792; doi:10.1093/infdis/jiz202)
Supplement: jiz202_suppl_Supplementary_Figures [file jiz202_suppl_supplementary_figures.docx]

Supplemental Fig. S1. Hematological Changes Following Challenge**.** Whole blood collected on Study Days 56 – 60 was analyzed for hematological changes using a HEMAVET® multispecies hematology instrument. Sham-vaccinated animals showed a significant reduction in white blood cells (WBCs) starting two days post-challenge compared to the MV-CHIK-vaccinated NHPs (p<0.05, unpaired t-test) as indicated by the asterisk. An ANOVA determined that there was no statistical difference between the time points for a given group (p>0.05). Bars indicate standard error.


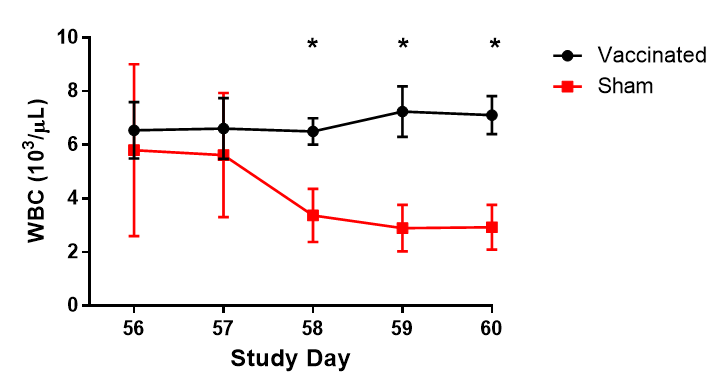


Supplemental Fig. S2. Viremia in cynomolgus macaques after challenge as measured by plaque assay. The limit of detection (100 pfu/ml) is indicated by the dashed line.
