# Supplementary material for: Immunogenicity and Efficacy of a Measles Virus-Vectored Chikungunya Vaccine in Nonhuman Primates
Source: J Infect Dis. 2019 May 3;220(5):735–42. doi: 10.1093/infdis/jiz202 (PMC6667792; doi:10.1093/infdis/jiz202)
Supplement: jiz202_suppl_Supplementary_Tables [file jiz202_suppl_supplementary_tables.docx]

###### Supplemental Table 1. Mean Hematology Values*

*If a sample as analyzed twice, the results from the second run were used in the mean calculations.

Abbreviations: WBC (white blood cell count, total), LY (lymphocytes), MO (monocytes), NE (neutrophils), EO (eosinophils), BA (basophils), LY# (lymphocyte count, total), MO# (monocyte count, total), NE# (neutrophils count, total), EO# (eosinophils count, total), BA# (basophils count, total), RBC (red blood cell count, total), Hgb (total hemoglobin), Hct (hematocrit), MCV (mean corpuscular volume), MCH (mean corpuscular hemoglobin), MCHC (mean corpuscular hemoglobin concentration), RDW (red cell distribution width), Plt (platelet count, total), MPV (mean platelet volume), SD (standard deviation).

###### Supplemental Table 2. Mean Clinical Chemistry Values

Abbreviations: GLU (glucose), BUN (blood urea nitrogen), CRE (creatinine), TBIL (total bilirubin), CA (calcium), PHOS (phosphate), ALB (albumin), TP (total protein), GLOB (globulin), ALT (alanine aminotransferase), ALP (alkaline phosphatase), AMY (amylase), NA+ (sodium), K+ (potassium), n/a (not applicable), SD (standard deviation)
